# Haemodynamic Monitoring Using Echocardiography in the Critically Ill: A Review

**DOI:** 10.1155/2012/139537

**Published:** 2012-02-14

**Authors:** Michelle S. Chew

**Affiliations:** Department of Intensive Care Medicine, Skåne University Hospital Malmö, Lund University, 20502 Malmö, Sweden

## Abstract

Physicians caring for the critically ill are now expected to acquire competence in echocardiography. It has become an indispensable diagnostic and monitoring tool in acute care settings where it is generally accepted to have therapeutic impact. There are a number of indications for a critical care echocardiographic study, and the most important queries include those pertaining left and right ventricular function and filling status. Focused examinations are increasing in popularity and provide a means for systematic study, and can be easily learned and practiced by novices. This paper addresses the indications, therapeutic impact, and some of the most common questions that can be answered using echocardiography the in critically ill patient.

## 1. Introduction

Echocardiography is now considered an indispensable tool for diagnosis and haemodynamic monitoring in critically ill patients. Indications for performing echocardiography in the ICU have expanded and it is now considered a requirement for critical care physicians to acquire competence in this mode of monitoring. Reflecting this are the numerous competency guidelines published in recent years [1–4].

Potential advantages and disadvantages of echocardiography compared to invasive haemodynamic monitoring (e.g., pulmonary artery catheter and arterial waveform analysis) in the critically ill are listed in Table 1.

This paper is not intended to be a comprehensive review of echocardiographic techniques. It does not include a review of left ventricular diastolic function, or lung ultrasound, a rapidly growing and increasingly important imaging modality [5].

Instead it addresses the indications, therapeutic impact, and some of the most common questions that can be answered using echocardiography in critically ill patients.

## 2. Therapeutic Impact

There are no randomized trials/metaanalyses regarding the impact of echocardiography on critically ill patients. A number of studies attest to the usefulness of echocardiography in the intensive care unit [6–9]. For example in Vignon et al., TTE and TEE led to therapeutic changes in approximately 25% of critically ill, mechanically ventilated patients [6], a finding supported by later studies [8, 9]. There are a number of societal guidelines with evidence-based recommendations for the use of echocardiography in a variety of clinical situations, including intraoperative settings and in critically ill patients [10]. The best evidence for the therapeutic impact of echocardiography in this context is found for perioperative TEE where improved clinical outcomes have been well documented [10].

## 3. Indications for Echocardiography in the Critically Ill

Echocardiography in critical care settings may be indicated for (1) diagnostic purposes, (2) guiding interventions and therapy, and (3) monitoring and followup.

The most important indications within the critical care context include diagnosis of major valvulopathies, major structural abnormalities (e.g., intracardiac masses, ventricular and atrial septal defects), endocarditis, pericardial effusion, and tamponade. It is also indicated for the evaluation of chest pain and unexplained shortness of breath, suspected pulmonary embolism, and respiratory failure of uncertain aetiology. It is used for the evaluation of shock or haemodynamic instability, where the determination of filling status and left and right ventricular function are key questions. In terms of monitoring, echocardiography may be used to assess responses to fluid and vasoactive therapies.

In the latest publication of the American College of Cardiology Appropriate Use Taskforce, appropriate use criteria were established for the use of TTE for cardiovascular evaluation in the acute care setting [11] (Table 2). Of note, assessment of volume status received an Appropriate Use Score of only 5 (of 9) points.

## 4. A Practical Approach

In recent years, several focused echocardiography protocols have been introduced [12]. These studies can usually be carried out by novice operators after a modest amount of training. For more complex examinations, consultation with the local echocardiography service is recommended if no specific competence is available in the intensive care unit.

There are several ways of approaching the echocardiographic examination of the critically ill. While several focused protocols exist, two such protocols, RACE and FATE, have gained widespread popularity and are described here.

This author finds RACE (rapid assessment by cardiac echo) useful for the initial echocardiographic evaluation of the unstable critically ill patient. This method ensures that the examination is conducted systematically, and stresses that findings be put within the context of the patient's clinical status. Two modes (M-mode and 2Dimensional imaging) and 5 views (parasternal long axis, parasternal short axis, apical 4-chamber, apical 2-chamber, and subcostal views) are used to answer the following four questions.

1. What is the left ventricular function?
2. What is the right ventricular function?
3. Is there any evidence of pericardial effusion and cardiac tamponade?
4. What is the fluid status?

The authors of RACE also stress that it is not a full TTE study, does not include Doppler measurements, and that a full transthoracic echocardiographic assessment should be requested if considered clinically necessary. Nevertheless, RACE is a good initial approach to the evaluation of the haemodynamically unstable patient and provides a skill set that can be easily learned by novices.

Another focused echocardiographic protocol is FATE (focused assessed transthoracic echocardiography) [13]. The purpose of FATE is to screen for significant pathology and to obtain information about volume status and cardiac contractility. FATE is similar to RACE in that it offers a systematic and focused approach to the echocardiographic examination of the critically ill patient, and provides a skill set that can be easily learned by novices. FATE differs from RACE in that it is not designed to answer a specific set of questions, and is rather used as a “rapid and systematic protocol for cardiopulmonary screening and monitoring” [13]. Another key difference is that in FATE other modalities such as Doppler may be applied as the user sees fit. Further, the examination may be interrupted before it is complete whereas RACE concentrates on answering the set of 4 questions systematically in every view.

## 5. Specific Areas of Interest in the Critically Ill

This paper will not include details of a full echocardiographic examination and the reader is referred instead to the numerous publications available with special focus areas [14–19]. However, a few key areas of interest to the critical care physician are outlined below. The importance of obtaining consistent and good quality images cannot be stressed enough. This is often a challenge in the critically ill, mechanically ventilated patient. Pathology should be confirmed from at least two views/windows. Less emphasis should be placed on obtaining direct measurements, for example, using Doppler methods due to the numerous associated pitfalls. The user is instead advised to conduct a systematic examination, obtain good quality images, and interpret the echocardiographic findings within the clinical context before embarking on various Doppler-based measurements.

### 5.1. LV Function

Assessment of global LV contractility may be quickly obtained by “eyeballing” from the parasternal long- and short-axis, apical 2- and 4-chamber and subcostal views [17, 18]. Experienced users may supplement this information by further assessments using a combination of ejection fraction/fractional shortening, Doppler patterns of ventricular filling, and tissue Doppler imaging [19]. It is important to use several windows as no single view can provide a comprehensive picture of contractility. In mechanically ventilated patients, obtaining parasternal views in particular may be challenging. In such patients, the subcostal view is often helpful since it minimizes signal attenuation from air in the lungs and the rib cage.

**Figure 1:**
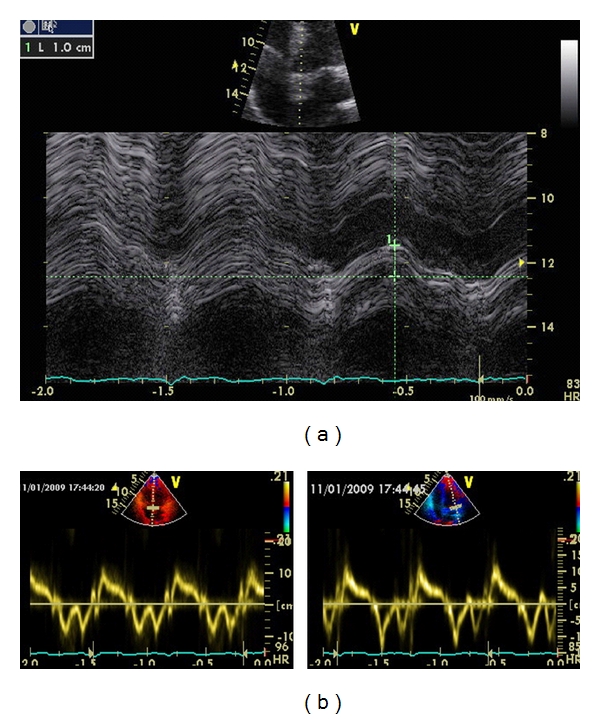
Methods for measuring LV function. (a) Atrioventricular plane displacement (septal wall) using M-mode, showing abnormal (decreased) displacement. (b) Systolic tissue Doppler measurement at the septal and lateral walls using tissue velocity imaging with pulsed wave Doppler, showing normal velocities.

Two other modes of imaging that are relatively easy to obtain for the assessment of LV function are the atrioventricular plane displacement (AVPD) and systolic tissue Doppler velocities (sTD) (Figure 1) [20–22]. Both of these are accessible from the apical window. Of note these measurements are dependent on preload, and only reflect components of LV contractility.

In addition to contractility, assessments of chamber size and LV wall thickness are made. These serve as an indication of fluid status, cardiomyopathies, and the presence of nonviable myocardium. Left atrial size is evaluated as an enlarged LA may indicate significant mitral and aortic valve disease, intra-atrial shunting and atrial fibrillation, all of which may contribute or cause haemodynamic instability. Further, LA size may provide an indication of elevated LV filling pressures.

Finally, the aortic and mitral valves are made to complete the examination of left ventricular function. Measurement of stenotic areas and regurgitant volumes are difficult and highly variable in the critically ill patients with varying volume status and mechanical ventilation. For this reason, echocardiographic evaluation of the critically ill should identify major pathology, but quantification of such should be made by experienced operators only, and taking into consideration the clinical context. A focused critical care echocardiographic examination should be able to identify, but not quantify, major valvulopathies that may contribute to or explain haemodynamic instability, such as significant aortic stenosis and mitral regurgitation, using 2D and colour Doppler imaging.

### 5.2. RV Function

Assessment of right ventricular function is of particular interest in critical care due to the effects of fluid loading and mechanical ventilation on the right heart. Due to ventricular interdependence [15], impaired RV function may lead to decreased left ventricular output. It is estimated that approximately 25% of patients with ARDS have right ventricular dysfunction and pulmonary hypertension [23]. Importantly, right ventricular failure is independently associated with mortality in critically ill patients [24].

**Figure 2:**
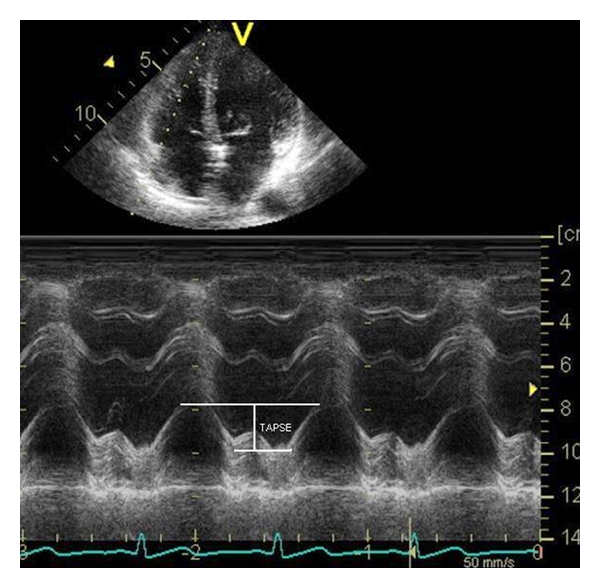
Tricuspid annular plane systolic excursion (TAPSE) for evaluating right ventricular contractility.

RV function is assessed initially from its size, wall thickness, and contractility. Comprehensive guidelines for the echocardiographic assessment of the right heart are given in a recent report of the American Society of Echocardiography [25]. For the critical care physician conducting an echocardiographic examination in mechanically ventilated patients, a more pragmatic approach may be adopted. Direct measurement of RV size by endocardial border tracing is difficult and not recommended due to its complex geometry and the presence of trabeculations within the RV chamber. Subjective assessment of the right ventricular area compared to left ventricular area in the apical 4-chamber view may be used instead. The RV should be smaller than the LV, and an RV : LV end diastolic area ratio of >0.6 indicates a dilated right ventricle, consistent with pressure or volume overload. Mechanical ventilation and pulmonary hypertension are common conditions causing RV dilatation in the critically ill patient. The right ventricular wall is normally thin, and hypertrophy indicates prior disease. RV contractility is assessed by eyeballing from the parasternal long-axis, apical 4-chamber, and subcostal views. Direct measurements such as the tricuspid annular plane systolic excursion (TAPSE) are easy to obtain and helpful, and provide a useful adjunct to eyeballing [25] (Figure 2).

The right atrium (RA) is examined for size and abnormal masses. A dilated RA may be indicative of fluid overload, interatrial shunts, tricuspid disease, and increased pulmonary pressures. Atrial fibrillation and mechanical ventilation may also cause a dilated RA. Finally the tricuspid and pulmonary valves are examined for abnormalities.

**Figure 3:**
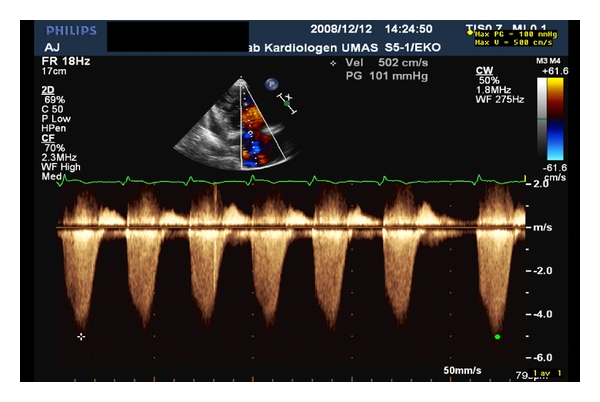
Estimation of the pulmonary arterial systolic pressure (PASP) from the tricuspid regurgitant jet (V_TR_). The latter is measured using continuous wave Doppler. PASP is calculated from simplified Bernoulli equation, PASP = 4 × V_TR_^2^.

Measurement of the tricuspid regurgitant velocity is a relatively simple procedure and is used for the estimation of pulmonary arterial systolic pressure using the simplified Bernoulli equation [12, 14]. Typically this is made from the apical 4-chamber view (Figure 3). If this is not accessible, the tricuspid regurgitant flow jet may also be insonated from the parasternal and subcostal views. Estimation of pulmonary arterial systolic pressure using this method assumes the absence of significant pulmonary stenosis, and may be inaccurate in patients with decreased right ventricular contractility.

### 5.3. Fluid Status

Estimation of preload by assessment of ventricular volumes is one of the most challenging areas in critical care echocardiography. Firstly, altering compliance complicates the pressure-volume relationship [26]. Added to this are the varying effects of mechanical ventilation on the heart. Generally preload assessment may be made by examination of the left ventricle, the right heart, and the inferior vena cava. The critical care physician may generally assess preload by measuring left ventricular volumes. The left ventricular end-diastolic area (LVEDA) may be “eyeballed” or measured using the Simpson's biplane method [27]. The latter requires identification of the endocardial border and may be difficult in the presence of mechanical ventilation. In the case of a hypovolaemic patient, a simpler approach is to look for obliteration of the LV cavity, also known as “kissing ventricles.”

The right ventricular dimensions are normally smaller than those of the LV. While RV dilatation may indicate volume overload, it is not specific for this. RV dilatation may occur for example due to mechanical ventilated with high PEEP. The RA size may be increased and an enlarged RA with bowing of the intra-atrial septum towards the left is indicative of elevated right atrial pressure. The triad of a “kissing” LV, small LV and RV size, along with a normal or small RA is strongly suggestive of hypovolaemia.

**Figure 4:**
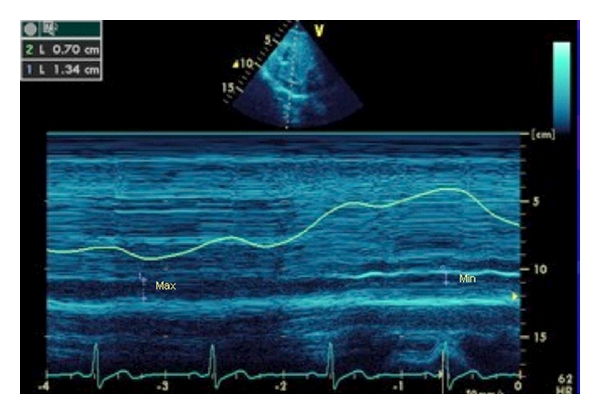
IVC diameter, measured using M-mode from the subcostal view. The minimum and maximum diameters are used to calculate the IVC distensibility and/or variability index. (Courtesy of A. McLean, S. Huang, and I. Ting, Nepean Critical Care Echo Group, Nepean Hospital, Sydney University, Australia).

A method for assessing fluid responsiveness in patients with controlled mechanical ventilation, that is, not on assist modes, is the distensibility index of the inferior vena cava (IVC_DI_). This is defined as

(1) $$\frac{D_{\max}-D_{\min}}{D_{\min}}\times 100\%,$$

where *D*_max⁡_ and *D*_min⁡_ are the minimum and maximum diameters of the inferior vena cava obtained from the subcostal view. A value exceeding 18% is predictive of fluid responsiveness in mechanically ventilated patients [28] (Figure 4). Another method which may be used is the variability index of the inferior vena cava (IVC_VI_) [29], defined as

(2) $$\frac{D_{\max}-D_{\min}}{D_{\text{mean}}},$$

where *D*_max⁡_ and *D*_min⁡_ are the minimum and maximum diameters of the inferior vena cava obtained from the subcostal view, and *D*_mean_ is the average of the two. A value >12% indicates fluid responsiveness in ventilated patients (Figure 4). 

IVC_DI_ and IVC_VI_ should be distinguished from the commonly used inferior vena cava collapsibility index, defined as *D*_max⁡_ − *D*_min⁡_/*D*_max⁡_. A small *D*_max⁡_ (<20 mm) with greater than 55% collapsibility is indicative of hypovolaemia [30]. However, this is relevant only in spontaneously breathing patients.

Finally the variation in the velocity time integral at the left ventricular outflow tract or aortic blood flow may predict volume responsiveness better than static indices. Generally thresholds around 15% have been shown to be predictive with sensitivities and specificities exceeding 90% [31–33].

### 5.4. Cardiac Output

**Figure 5:**
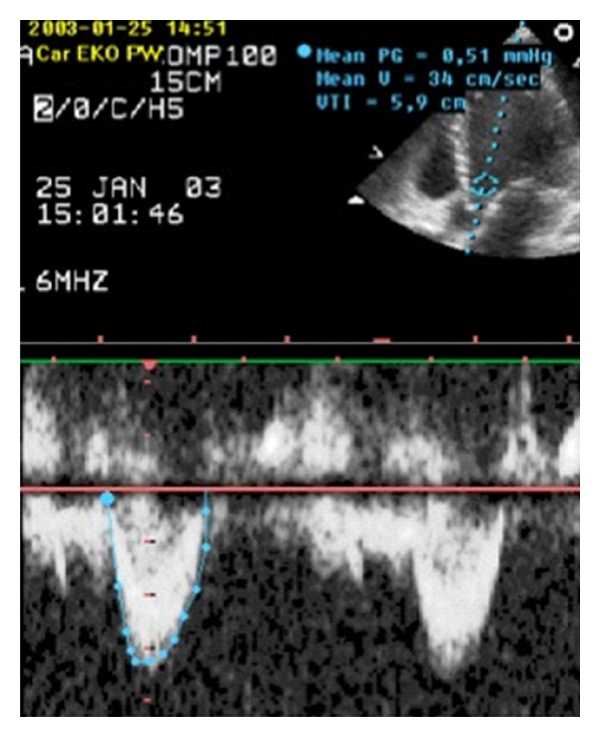
Measurement of LVOT VTI from the apical 5-chamber plane.

Cardiac output (CO) measurements are occasionally made in the critical care setting, since an adequate CO is a prerequisite for tissue oxygen delivery. While a low CO is always a source of concern, there is no pre-set absolute value for adequate CO. Hence in some situations a “high” CO of 10 L/min may be adequate, and conversely a seemingly “normal” CO of 5 L/min may be inadequate for optimal tissue oxygen delivery. There are several ways of measuring CO echocardiographically. One commonly used and reliable method relies on the measurement of the velocity time integral from the left ventricular outflow tract (LVOT VTI) in an apical 5-chamber plane (Figure 5) [26, 34]. The diameter of the aortic annulus is measured from the parasternal long-axis view, and its area was calculated. Multiplying this area with the LVOT VTI gives the stroke volume, and multiplying stroke volume with heart rate gives the CO.

### 5.5. Pericardial Effusion and Tamponade

Echocardiography is the tool of choice for evaluating the pericardial sac and the presence of tamponade. The diagnosis of a pericardial effusion is made from the observation of an echo-free space between the parietal and visceral pericardium seen from the parasternal, apical, and/or subcostal views.

The presence of haemodynamically significant pericardial fluid is typically assessed by examination of the RA and RV. RA collapse during early systole and RV collapse during early diastole indicate that intrapericardial pressure exceeds right heart pressures. These findings, together with a dilated IVC are signs of a haemodynamically significant tamponade [35].

## 6. Conclusion

Echocardiography is important development in critical care. However, as with any diagnostic and monitoring tool, echocardiography is subject to errors in interpretation, and there is a range of individual responses for any given study. No single tool is complete; however, echo provides some distinct advantages compared to invasive monitoring, not least of which are noninvasiveness and the ability to conduct a direct anatomic evaluation of the heart and its component parts in real time.

There are a number of focused approaches designed to facilitate the conduct of a systematic echocardiographic study. A number of guidelines have been issued for training and competency, which are designed to enforce standards and define core skill sets required for examination of the critically ill patient. The most important of these have been addressed in this review.

## Figures and Tables

**Table 1:** Potential advantages and disadvantages of echocardiography versus invasive monitoring.

	Echocardiography	Invasive haemodynamic monitoring
Invasiveness	TTE noninvasive TEE semi-invasive	PAC invasive arterial waveform analysis semi-invasive

Portability	Scanners easily moved to patient	Generally not portable

Use in acute care	Yes, also documented for ED	No

Diagnostic value	Yes	Yes

Monitoring capability	Yes	Yes

User dependent	Very user dependent	Less user dependent, some methods require calibration

PAC: pulmonary artery catheter; TTE: transthoracic echocardiography; TEE: transoesophageal echocardiography.

**Table 2:** Indications for echocardiography in acute care settings, evaluated using appropriate use scores (AUS).

Indication	AUS
Hypotension/haemodynamic instability of uncertain or suspected cardiac aetiology	A

Assessment of volume status in critically ill patient	U

Acute chest pain with suspected MI, inconclusive ECG during pain	A

No chest pain but laboratory and/or other features indicative of MI	A

Suspected complication of MI	A

Respiratory failure/hypoxemia of uncertain aetiology	A

Respiratory failure/hypoxemia when noncardiac aetiology is already established	U

To establish diagnosis of suspected PE	I

To guide therapy of known acute PE	A

Routine surveillance of prior PE, with normal RV function and PAP	I

Reevaluation of known PE after therapy for change RV function and PAP	A

Severe deceleration injury/chest trauma with suspected or possible pericardial effusion, valvular, or cardiac injury	A

Routine evaluation in mild chest trauma without ECG or biomarker changes	I

I: inappropriate test for that indication (not generally acceptable and not a reasonable approach. Score 1–3 out of 9); U: uncertain for specific indication (may be acceptable and may be a reasonable approach. Also implies that further patient information/research needed to classify indication definitively. Score 4–6 out of 9); A: appropriate test for that indication. Test is generally acceptable and is a reasonable approach for the indication. Score 4–6 out of 9). MI: myocardial infarction, PE: pulmonary embolism, RV: right ventricle, PAP: pulmonary arterial pressure. Adapted from Douglas et al. [11].
